# Efficacy of a topical formulation containing esafoxolaner, eprinomectin and praziquantel (NexGard Combo^®^) against natural infestations with the cat louse, *Felicola subrostratus* under field conditions[Fn FN1]

**DOI:** 10.1051/parasite/2022062

**Published:** 2022-12-23

**Authors:** Andrei Daniel Mihalca, Georgiana Deak, Luciana Cătălina Panait, Ștefan Rabei, Frederic Beugnet

**Affiliations:** 1 Department of Parasitology and Parasitic Diseases, University of Agricultural Sciences and Veterinary Medicine of Cluj-Napoca Calea Mănăștur 3–5 400372 Cluj-Napoca Romania; 2 Parasitology Consultancy Group SRL Strada Principală 145B 407056 Corușu Romania; 3 Boehringer Ingelheim Animal Health Av Tony Garnier 69007 Lyon France

**Keywords:** Cat, Chewing lice, *Felicola subrostratus*, Esafoxolaner, Efficacy assessment

## Abstract

*Felicola subrostratus* is the only species of louse affecting domestic cats. Although it is considered a rare ectoparasite of pet cats, it occurs mainly in stray or shelter animals and is sometimes associated with intense pruritus and secondary bacterial infections. The aim of the present study was to evaluate the efficacy of a single dose of the topical formulation of esafoxolaner, eprinomectin and praziquantel (NexGard Combo^®^, Boehringer Ingelheim) in cats for the treatment of naturally acquired chewing lice infestation in a multi-site, positive-control, blinded clinical field study. Thirty-one domestic cats presenting natural *F. subrostratus* infestation were included in the study. The animals had not been treated with any ectoparasiticide within the previous 60 days. After inclusion, each cat was randomly assigned to one of the two groups: group 1, 14 cats treated with NexGard Combo^®^ or group 2, 17 cats treated with Frontline Combo^®^. A clinical evaluation was performed at days 0 (inclusion), 14 and 30 and consisted in scoring the skin lesions and symptoms and scoring the presence of lice. On day 30, all cats from both groups scored 0 for the presence of live lice and no dead lice were found, demonstrating 100% efficacy. The clinical scores significantly improved from day 0 to day 30 in both groups. During the 30 days of surveillance, no reinfestations due to the hatching of eggs were observed and none of the cats had any adverse reactions. Esafoxolaner demonstrated high efficacy for the treatment of *F. subrostratus* infestation.

## Abstract (fr)

*Felicola subrostratus* est la seule espèce de pou affectant les chats domestiques. Bien que ce pou soit rare comme ectoparasite chez les chats de compagnie, il infeste principalement les animaux errants ou en refuge et est parfois associé à un prurit intense et à des infections bactériennes secondaires. Le but de la présente étude était d’évaluer l’efficacité d’une dose unique de la formulation topique d’esafoxolaner, d’éprinomectine et de praziquantel (NexGard Combo^®^, Boehringer Ingelheim) chez le chat pour le traitement de l’infestation naturelle par les poux broyeurs dans une étude de terrain multi-sites, randomisée, avec un contrôle positif. Trente et un chats domestiques présentant une infestation naturelle par *F. subrostratus* ont été inclus dans l’étude. Les animaux n’avaient été traités avec aucun ectoparasiticide au cours des 60 jours précédents. Après inclusion, chaque chat a été assigné au hasard à l’un des deux groupes : groupe 1, 14 chats traités avec NexGard Combo^®^, ou groupe 2, 17 chats traités avec Frontline Combo^®^. Une évaluation clinique a été réalisée aux jours 0 (inclusion), 14 et 30 et a consisté à un score clinique des lésions et symptômes cutanés et à noter la présence de poux. Au jour 30, tous les chats des deux groupes ont obtenu un score de 0 pour la présence de poux vivants et aucun pou mort n’a été trouvé, démontrant une efficacité de 100 %. Les scores cliniques se sont significativement améliorés du jour 0 au jour 30 dans les deux groupes. Au cours des 30 jours de surveillance, aucune réinfestation due à l’éclosion des œufs n’a été observée et aucun des chats n’a présenté d’effet indésirable. L’esafoxolaner a démontré une efficacité élevée pour le traitement de l’infestation par *F. subrostratus*.

## Introduction

*Felicola subrostratus* (Phthiraptera, Mallophaga, Trichodectidae) is a species of chewing louse (also known as biting louse) affecting domestic cats worldwide and occasionally reported from several species of wild felids [5, 14]. It is the only louse affecting domestic cats [15] and being highly host specific, they do not infest other mammals such as dogs or humans [3]. *Felicola subrostratus* is considered a rare ectoparasite of pet cats, but it is common in stray or shelter animals [3]. *Felicola subrostratus* seems to have a cosmopolitan distribution with sporadic reports from North America (United States, Mexico), Central America (Panama), South America (Uruguay, Brazil), the Caribbean (Cuba), Pacific Islands (Guam), Australia, Asia (Israel, Turkey, Malaysia, Thailand) and Europe (Greece, Albania, Hungary, Italy, Slovenia) with European prevalence rates between 0.5% and 8.3% [1, 4].

The clinical picture associated with the infestation by *F. subrostratus* ranges from asymptomatic infestations to intense pruritus and secondary bacterial infections. Clinical signs and intensity of the infestation are generally more common in old, debilitated or chronically ill cats, and mainly those with long hair, due to deficient or absence of grooming [15, 17, 19]. There is limited data on the vectorial role of *F. subrostratus* with only a potential role as intermediate host for *Dipylidium* spp. [11].

Due to the detrimental impact on the health of cats as well as to break the transmission cycle, all confirmed infestations with *F. subrostratus* should be treated. To date, only a few clinical studies have evaluated the efficacy of insecticides against *F. subrostratus* in cats, and spot-on or spray-based drugs such as fipronil [13] or selamectin [6] have been demonstrated to be effective after a single topical administration (Table 1). Recently, afoxolaner has been demonstrated to be highly effective against the canine chewing lice, *Trichodectes canis* [12]. However, no studies are available to evaluate the efficacy of isoxazolines against *F. subrostratus* in cats. The first isoxazolines available on the veterinary pharmaceutical market were the oral chewables containing afoxolaner and fluralaner in 2014. They were shortly followed by sarolaner in 2015, and lotilaner in 2017 [7]. Isoxazolines are considered broad-spectrum and safe ectoparasiticides for pets and are efficient in treating of infestations with various arthropods groups (ticks, mites, fleas, and lice) [10, 12, 22]. Moreover, there is no resistance reported for isoxazolines. Isoxazolines act as inhibitors of the helical subunits of Gamma-aminobutyric acid (GABA), a neurotransmitter found in the peripheral nervous system of invertebrates, and have a strong inhibitory activity on the glutamate-gated chloride channel in invertebrates [22].

**Table 1:** Overview of the efficacy field studies of various antiparasitic products against *Felicola subrostratus.*

Compound(s)	Formulations	Days of follow-up	Efficacy (%)	Reference
Fipronil	Spray	2, 28, 42	98.2–100^1^	[13]
Fipronil	Spot-on	2, 28, 42	98.3–100^1^	[13]
Propoxur	Collar	2, 28, 42	100^2^	[13]
Selamectin	Spot-on	7, 14, 21, 28, 35, 42	100^3^	[6]
Esafoxolaner	Oral	14, 30	92.9–100^4^	Current study

1 98.2% efficacy at day 0, 100% efficacy at days 28 and 42;

2 At all follow-ups;

3 At all follow ups;

4 92.2% at 14 days, 100% at 30 days post-treatment.

The aim of the present study was to determine the efficacy of a single dose of the topical formulation of esafoxolaner, eprinomectin and praziquantel (NexGard Combo^®^, Boehringer Ingelheim) in cats for the treatment of naturally acquired chewing lice infestation under field conditions [2].

## Materials and methods

### Study site and included animals

The multi-site, positive-control, blinded clinical efficacy field study was implemented in the region of Transylvania, Romania. Between 26 August 2021 and 23 February 2022, 31 domestic cats (15 females, 16 males), aged between 2 months and 10 years (8 cats <6 months, 3 cats 6–12 months, 7 cats 12–24 months, 13 cats >24 months) were included in the study (see inclusion and exclusion criteria below). The cats originated from 6 counties (9 localities), as follows: Alba (Roșia Montană – 1 cat), Bihor (Oradea – 1 cat), Bistrița-Năsăud (Beudiu – 5 cats, Malin – 6 cats, Nușeni – 10 cats, Rusu de Sus – 4 cats), Braşov (Braşov – 2 cats), Maramureș (Baia Mare – 1 cat), and Sibiu (Săcădate – 1 cat) (All details included in supplementary file). One cat originally included in the study was removed as it disappeared during the study.

### Inclusion, exclusion and removal criteria

For inclusion, the body surface of privately owned cats was carefully inspected for the presence of chewing lice, *F. subrostratus*. Only infested cats (presence of adult motile stages and at least 1 nit), clinically healthy (except the skin lesions consistent with mallophagosis such as pruritus, hair loss, and presence of scales), with a weight of at least 2 kg, and an age of at least 8 weeks, were included. The animals had not been treated with any ectoparasiticide within the previous 60 days. The aim and study design were explained to the owners, and they were asked to sign an informed consent.

After visual confirmation of the presence of lice, one adult louse was collected in ethanol from each animal and later confirmed microscopically as *F. subrostratus*, according to morphological criteria [7].

Exclusion criteria were: presence of other clinical signs than those consistent with the presence of chewing lice, animals that had been treated with topical or systemic ectoparasiticides within the last three months or within the efficacy duration of the respective ectoparasiticide drug, pregnant or lactating females and females intended for breeding during the study.

After inclusion, cats lost or disappeared, cats who changed owner, owners who withdrew consent, cats with inappropriate health status or behaviour in the context of the study, or cats from sites that had been treated with environmental ectoparasiticides after day 0 were excluded.

### Randomization, study groups, treatment, and evaluation

After inclusion, each cat was randomly assigned to one of the two study groups: group 1 – treated with NexGard Combo^®^ according to the dosing table from the product label or group 2 – reference positive control treated with Frontline Combo^®^ cat according to the label instructions. If several cats from the same household were included in the study, they were all allocated to the same study group. If more cats were present in the same household, they were all treated with the same product as the included cat(s) even in the absence of chewing lice infestation.

The clinical evaluation was done on days 0 (inclusion), 14 and 30 and consisted in recording and scoring the skin lesions and symptoms: pruritus (0 = absent; 1 = mild without alteration of the skin; 2 = moderate with mild alterations of the skin; 3 = severe with pronounced alterations of the skin), hair loss (0 = absent; 1 = very limited; 2 = mild; 3 = extensive), and presence of scales (0 = absent; 1 = very limited; 2 = mild; 3 = extensive).

A scoring system was applied for grading the level of cat lice infestation: 1 (nits + 1 adult chewing louse), 2 (nits + <10 chewing lice), or 3 (nits + >10 chewing lice). No distinction was made between nymphs and adults during the lice count. The presence of other ectoparasites (fleas, ticks, or ear mites) was also recorded. No skin scrapings were performed.

### Statistical analysis

Statistical analyses were performed separately for the group tested with NexGard Combo^®^ and the reference positive control group. Statistical associations between categorical variables, such as days 0, 14 and 30 and the scores used to assess pruritus, hair loss and the presence of scales and lice, were evaluated using the non-parametric Wilcoxon signed-rank test. Results were considered statistically significant at a *p-*value < 0.05. Data analyses were performed using R software v. 4.0.5 (R Foundation for Statistical Computing, Vienna, Austria).

## Results

Thirty-one cats completed the study. Fourteen were assigned to group 1 (treated with NexGard Combo^®^) and 17 were assigned to group 2 (positive control, treated with Frontline Combo^®^ cat).

**Figure 1:**
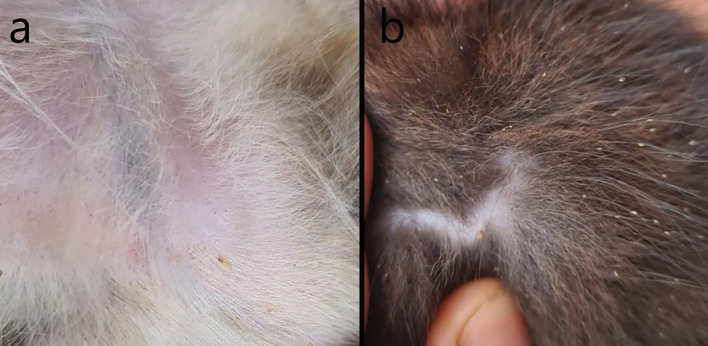
Infestation with *Felicola subrostratus* in domestic cats. (a) Note the adult louse; (b) presence of adult louse and nits.

The lice infestation scores on day 0 were 2 or 3 (Fig. 1) (Table 2). On day 14, 30 cats scored 0 for lice and one cat from group 1 still scored 3. On day 14, 2/14 group 1 cats still had nits. In group 2, nits were found on day 14 on 1 cat. On day 30, all cats from both groups scored 0 for the presence of alive lice. Two cats from group 1 still had nits. No cats from group 2 had nits. During the 30 days of surveillance, no reinfestations due to the potential hatching of eggs were observed. The lice score decrease was significant in both groups from day 0 to day 30. These results demonstrate a parasiticidal efficacy of 100% at 30 days in both treatment groups. During the evaluation period, none of the cats had any adverse reactions.

**Table 2:** Day 0 scoring of lice infestation in the cats from the two groups.

Groups	Score 2 (*n*)	Score 3 (*n*)	Total
Group 1	5	9	14
Group 2	2	15	17
Total	7	24	31

**Figure 2:**
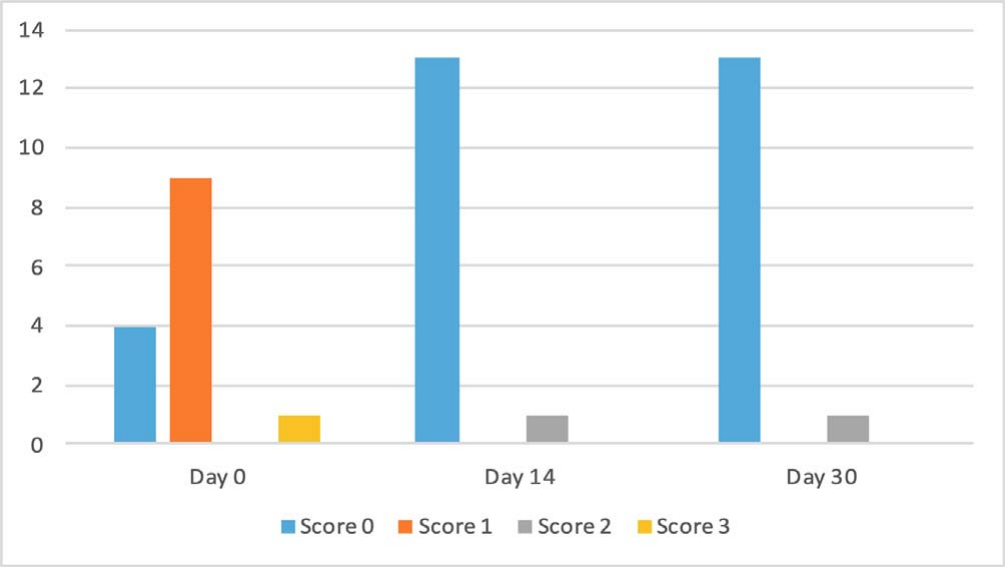
Number of cats according to the clinical score for pruritus in Group 1 (NexGard Combo^®^).

**Figure 3:**
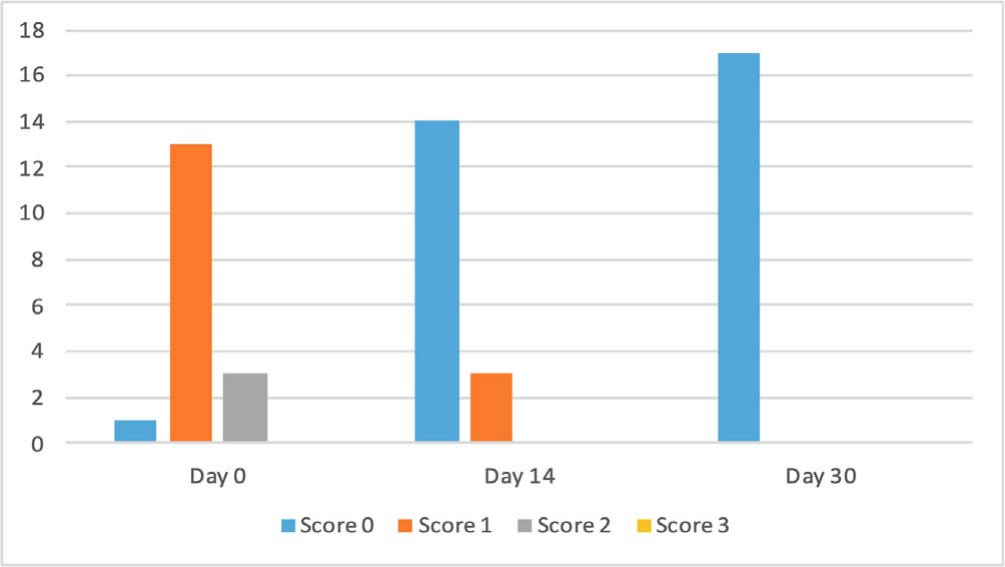
Number of cats according to the clinical score for pruritus in Group 2 (Frontline Combo^®^).

**Figure 4:**
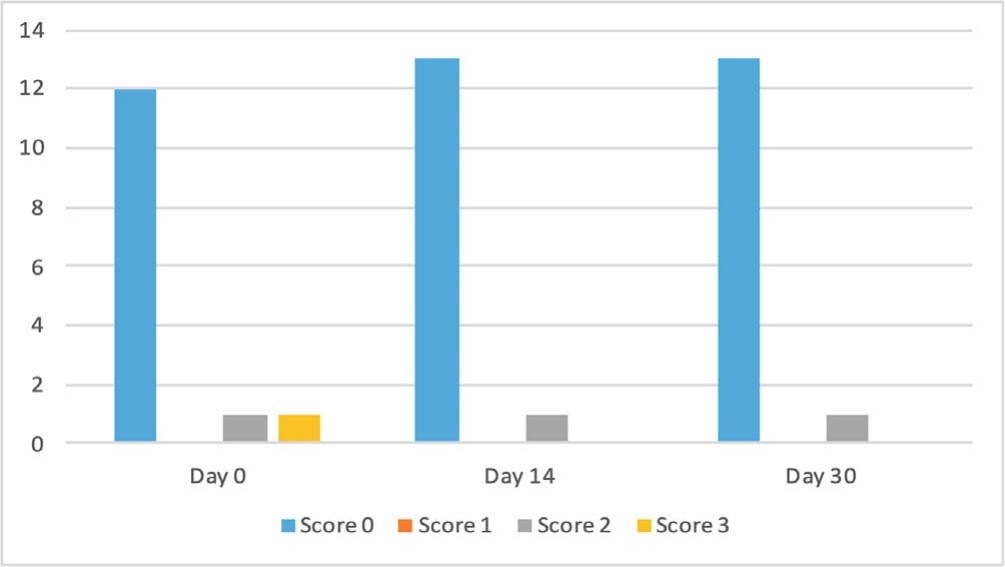
Number of cats according to the clinical score for hair loss in Group 1 (NexGard Combo^®^).

**Figure 5:**
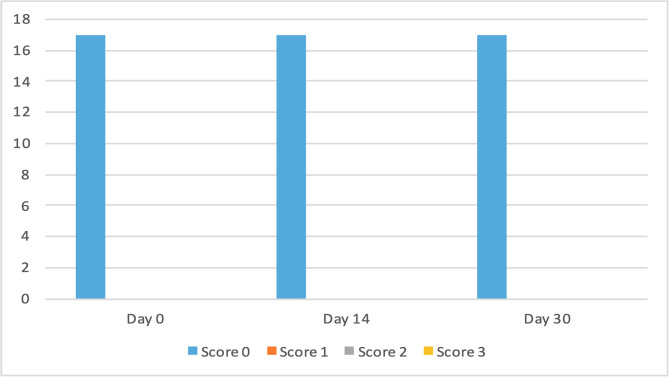
Number of cats according to the clinical score for hair loss in Group 2 (Frontline Combo^®^).

**Figure 6:**
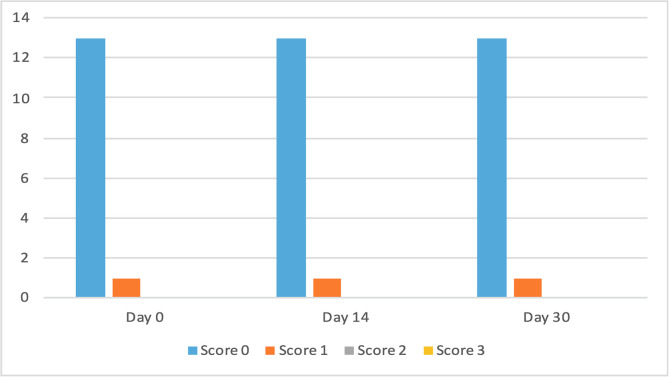
Number of cats according to the clinical score for scales in Group 1 (NexGard Combo^®^).

**Figure 7:**
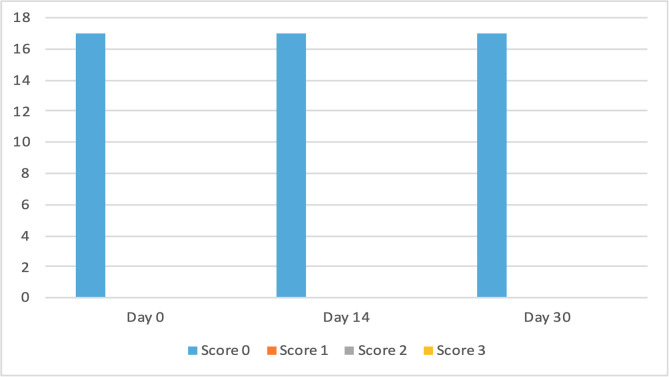
Number of cats according to the clinical score for scales in Group 2 (Frontline Combo^®^).

**Figure 8:**
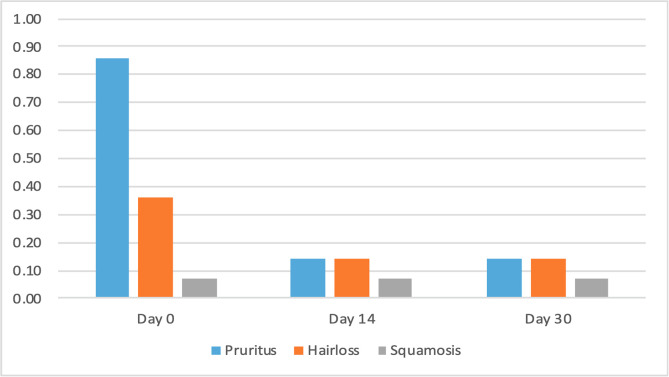
Average score for clinical signs associated with lice in cats from Group 1 (NexGard Combo^®^).

**Figure 9:**
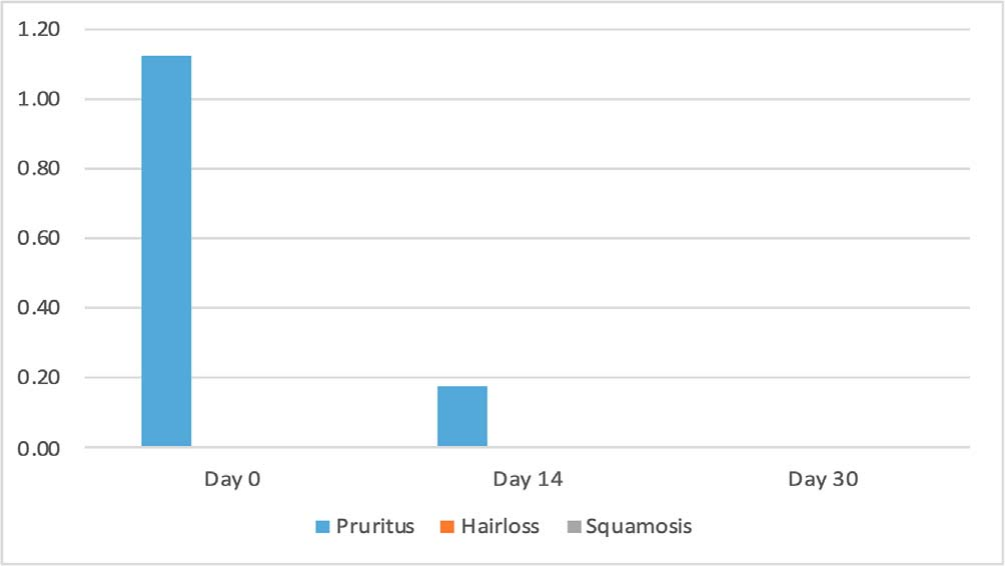
Average score for clinical signs associated with lice in cats from Group 2 (Frontline Combo^®^).

The clinical scores for evaluated dermatological signs improved in both groups on day 14 and day 30 (Figures 2–9). A statistically significant decrease in pruritus, hair loss and the presence of scales and lice was observed in both groups following treatment (*p* < 0.001). No significant differences were observed between the two treatment groups.

On day 0, 7 of the 31 cats were also infested with other ectoparasites (i.e., fleas and/or *Otodectes* – not collected, not identified to species level). At day 14 and day 30, these other ectoparasites were not found.

## Discussion

The study demonstrated high efficacy of an isoxazoline, esafoxolaner, against *F. subrostratus* in cats, and it is the first of its kind for this parasite. The topical formulation fipronil – (S)-methoprene was chosen as positive control because the indication against cat chewing lice is registered in Europe. Similarly, a recent study demonstrated the efficacy of afoxolaner against *T. canis* in dogs [12]. As previously shown [12], off-label afoxolaner was demonstrated to have a very high to maximum efficacy also against lice in birds [20, 21]. Another isoxazoline, fluralaner presented an efficacy of 85.1% to 100% against *Linognathus setosus* in dogs with follow-ups done on days 1, 7, 28 and 84 [10].

Isoxazolines act through a systemic pathway [16]. Esafoxolaner is absorbed transcutaneously when administered topically and is highly bound to plasma proteins [8]. Such isooxazoline systemic formulations are firstly intended to act against hematophagous parasites (i.e., fleas and ticks) [14]. Besides fleas and ticks, esafoxolaner has demonstrated its efficacy against mites (i.e., *Otodectes cynotis* and *Notoedres cati*) which induce inflammatory reactions [9, 18]. It can be hypothesized that esafoxolaner is present in inflammatory fluids as in plasma. Regarding chewing lice, they are more superficial, but they induce a skin reaction which is sufficient for *Trichodectes* and *Felicola* lice to ingest afoxolaner or esafoxolaner, respectively, and to be killed.

## Conclusion

In conclusion, esafoxolaner demonstrated high efficacy for the treatment of the feline chewing louse *F. subrostratus* infestation, adding another feline ectoparasite to its broad spectrum. Esafoxolaner (NexGard Combo^®^) is approved (per label in various countries) for the treatment of a wide range of ectoparasites in domestic cats such as *Ctenocephalides felis*, *Ixodes scapularis*, *I. ricinus*, *I. holocyclus*, *Dermacentor variabilis*, *Haemaphysalis longicornis*, *Otodectes cynotis* and *Notoedres cati* [22].

## Supplementary material

The supplementary material of this article is available at https://www.parasite-journal.org/10.1051/parasite/2022016/olm. Special Issue - NexGard® Combo supplementary material
